# Gut dysbiosis is associated with metabolism and systemic inflammation in patients with ischemic stroke

**DOI:** 10.1371/journal.pone.0171521

**Published:** 2017-02-06

**Authors:** Kazuo Yamashiro, Ryota Tanaka, Takao Urabe, Yuji Ueno, Yuichiro Yamashiro, Koji Nomoto, Takuya Takahashi, Hirokazu Tsuji, Takashi Asahara, Nobutaka Hattori

**Affiliations:** 1 Department of Neurology, Juntendo University School of Medicine, Tokyo, Japan; 2 Department of Neurology, Juntendo University Urayasu Hospital, Chiba, Japan; 3 Probiotics Research Laboratory, Juntendo University Graduate School of Medicine, Tokyo, Japan; 4 Yakult Central Institute, Tokyo, Japan; Wageningen University, NETHERLANDS

## Abstract

The role of metabolic diseases in ischemic stroke has become a primary concern in both research and clinical practice. Increasing evidence suggests that dysbiosis is associated with metabolic diseases. The aim of this study was to investigate whether the gut microbiota, as well as concentrations of organic acids, the major products of dietary fiber fermentation by the gut microbiota, are altered in patients with ischemic stroke, and to examine the association between these changes and host metabolism and inflammation. We analyzed the composition of the fecal gut microbiota and the concentrations of fecal organic acids in 41 ischemic stroke patients and 40 control subjects via 16S and 23S rRNA-targeted quantitative reverse transcription (qRT)-PCR and high-performance liquid chromatography analyses, respectively. Multivariable linear regression analysis was subsequently performed to evaluate the relationships between ischemic stroke and bacterial counts and organic acid concentrations. Correlations between bioclinical markers and bacterial counts and organic acids concentrations were also evaluated. Although only the bacterial counts of *Lactobacillus ruminis* were significantly higher in stroke patients compared to controls, multivariable analysis showed that ischemic stroke was independently associated with increased bacterial counts of *Atopobium* cluster and *Lactobacillus ruminis*, and decreased numbers of *Lactobacillus sakei* subgroup, independent of age, hypertension, and type 2 diabetes. Changes in the prevalence of *Lactobacillus ruminis* were positively correlated with serum interleukin-6 levels. In addition, ischemic stroke was associated with decreased and increased concentrations of acetic acid and valeric acid, respectively. Meanwhile, changes in acetic acid concentrations were negatively correlated with the levels of glycated hemoglobin and low-density lipoprotein cholesterol, whereas changes in valeric acid concentrations were positively correlated with the level of high sensitivity C-reactive protein and with white blood cell counts. Together, our findings suggest that gut dysbiosis in patients with ischemic stroke is associated with host metabolism and inflammation.

## Introduction

Ischemic stroke is associated with metabolic diseases including obesity, type 2 diabetes (T2D), and dyslipidemia. Systemic low-grade inflammation is also closely linked to metabolic disorders [1] and plays a substantial role in the pathogenesis of cardiovascular diseases, including ischemic stroke [2, 3]. As the prevalence of metabolic diseases has continued to increase over the past decades [4–6], their role in ischemic stroke has become more relevant [7, 8].

Increasing evidence suggests that dysbiosis of the gut microbiota is associated with the pathogenesis of both intestinal disorders, such as inflammatory bowel disease, and extra-intestinal disorders, including metabolic diseases [9]. Alterations in the composition of the gut microbiota have been reported in individuals with obesity [10–11] and T2D [12–14]. In addition, trimethylamine-*N*-oxide (TMAO), a metabolite of the gut microbiota, was shown to promote atherosclerosis [15, 16]. However, whereas the blood TMAO level was reported to predict cardiovascular disease risk [17], a recent study found that the blood TMAO level in patients with stroke and transient ischemic attack was lower, rather than higher, than that of the asymptomatic group [18].

In particular, individuals with obesity [11] and T2D [13] exhibit changes in the populations of bacteria that produce organic acids (primarily acetic, propionic, and butyric acid), which represent the primary products of dietary fiber fermentation by the gut microbiota and comprise key pathophysiological molecules for modulating host inflammation and metabolism [19, 20]. Furthermore, in patients with symptomatic carotid atherosclerosis, gut metagenomes were enriched in genes encoding the peptidoglycan pathway and depleted in genes involved in the synthesis of anti-inflammatory molecules and antioxidants, suggesting an association between gut metagenomes and host inflammatory status [21].

The purpose of the present study was to determine whether the composition of the gut microbiota and fecal organic acid concentrations are associated with ischemic stroke and, if so, to examine the associations between these changes and host metabolism and inflammation.

## Materials and methods

### Subjects

Patients with acute ischemic stroke (n = 175) who were admitted to the Department of Neurology, Juntendo University Hospital, between April 2014 and March 2015, were examined as potential study participants. The primary inclusion criterion was acute ischemic stroke diagnosed by neurologists. All patients underwent brain magnetic resonance imaging or computed tomography to evaluate ischemic lesions. Exclusion criteria included: admission > 24 h from stroke onset (n = 37); dissection-associated stroke (n = 6), patent foramen ovale (n = 5), or vasculitis (n = 1); a history of malignancy (n = 13), autoimmune disease (n = 6), chronic kidney disease with hemodialysis (n = 6), Parkinson’s disease (n = 5), or valvular replacement (n = 3); prior intravenous thrombolysis treatment (n = 14); and non-availability of fecal samples (n = 38). In total, 41 patients were included in the study cohort. The stroke subtypes, according to the TOAST classification system [22], were large-artery atherosclerosis (n = 8; 20%), cardioembolism (n = 4; 10%), small-vessel occlusion (n = 10; 24%), other or undetermined etiology (n = 17; 41%), and transient ischemic attack (n = 2; 5%). Cases of stroke of other or undetermined etiology included aortogenic embolism (n = 2), branch atheromatous disease (n = 12), and embolic stroke of undetermined source (n = 3). The mean National Institutes of Health Stroke Scale (NIHSS) on admission was 3.2 ± 2.8 (range, 0 to 16). We also recruited age- and sex-matched control subjects (n = 40) among individuals who regularly visited our outpatient clinic for the management of hypertension, dyslipidemia, or chronic headache between June 2014 and February 2015. All subjects were in good physical condition. All control subjects lacked a history of stroke, coronary artery disease, peripheral artery disease, malignancy, autoimmune disease, or neurodegenerative disease. Neither stroke patients nor controls had a history of intestinal disorders or had been treated with antibiotics in the 2 months prior to inclusion.

This study was approved by the local ethics committee at the Juntendo University School of Medicine, Japan, in accordance with the ethical standards of the 1964 Declaration of Helsinki and its later amendments. Written informed consent was obtained from all participants.

### Biochemical assays

Blood samples were obtained from patients with stroke at admission and from control subjects at the outpatient clinic. Serum levels of glycated hemoglobin (HbA1c), high-density lipoprotein (HDL) cholesterol, low-density lipoprotein (LDL) cholesterol, and triglycerides (TG) were measured using standard techniques. The plasma levels of high-sensitivity C-reactive protein (hsCRP), interleukin (IL)-6, and tumor necrosis factor (TNF)-α were measured by latex nephelometry, chemiluminescent enzyme immunoassay, and enzyme-linked immunosorbent assay (ELISA) analysis, respectively at a private laboratory facility (SRL Diagnostics, Tokyo, Japan). Plasma levels of lipopolysaccharide-binding protein (LBP) were measured using a human LBP ELISA kit (Hycult Biotech, Uden, The Netherlands).

### 16S and 23S rRNA-targeted quantitative reverse transcription (qRT)-PCR

After enrollment, participants were asked to submit fresh fecal samples. None of the subjects were administered antibiotics during the collection period. The first available fecal samples from stroke patients were collected by hospital staff and placed directly into two tubes (approximately 1.0 g/tube); one for organic acid measurement and the other containing 2 ml RNAlater (Ambion, Austin, TX, USA) for bacterial analysis. The mean duration between admission and fecal sample collection was 3.8 ± 2.0 days. Fecal samples were stored at -20°C immediately after collection. Control subjects were given materials and instructions for collecting fecal samples at home. Samples were kept at -20°C in a cooling box with refrigerants and sent to Juntendo University. Fecal samples were obtained within 1 week following visits to the outpatient clinic. For the analysis of bacteria in blood samples, 1 ml blood was added to 2 ml RNAprotect Bacteria Reagent (Qiagen, Venlo, Netherlands) immediately after collection and stored at -80°C. Both fecal and blood samples were transported at -20°C to the Yakult Central Institute, and total RNA was extracted as previously described [23–25].

To examine the gut microbial composition and blood levels of gut bacteria, targeted 16S and 23S rRNA qRT-PCR was conducted using the Yakult Intestinal Flora-SCAN analysis system (YIF-SCAN^®^, Yakult Honsha Co., Ltd., Tokyo, Japan). Three serial dilutions of each extracted RNA sample were used for qRT-PCR analysis, and threshold cycle values within the linear range of the assay were applied to a standard curve to obtain corresponding bacterial cell counts for each fecal or blood sample. The specificity of the qRT-PCR assay was assessed using group-, genus-, and species-specific primers, respectively, as described previously [23–25].

In our study, the 22 bacterial groups/genera/species examined were comprised of (1) six anaerobes that predominate the human intestine (*Clostridium coccoides* group, *Clostridium leptum* subgroup, *Bacteroides fragilis* group, *Bifidobacterium*, *Atopobium* cluster, and *Prevotella*); (2) seven potential pathogens (*Clostridium difficile*, *Clostridium perfringens*, *Enterobacteriaceae*, *Enterococcus* spp., *Streptococcus* spp., *Staphylococcus* spp., and *Pseudomonas* spp.); and (3) nine lactobacilli (*L*. *gasseri* subgroup, *L*. *brevis*, *L*. *casei* subgroup, *L*. *fermentum*, *L*. *fructivorans*, *L*. *plantarum* subgroup, *L*. *reuteri* subgroup, *L*. *ruminis* subgroup, and *L*. *sakei* subgroup). The sequences of the primers used for these analyses are listed in S1 Table.

### Measurement of organic acid concentrations and pH

Fecal organic acid concentrations were determined as described previously [26], but with slight modifications. Briefly, frozen samples were homogenized in a four-fold volume of 0.15 mol/l perchloric acid, maintained at 4°C for 12 h, then centrifuged at 20,400 × *g* at 4°C for 10 min. The resulting supernatants were then passed through a 0.45-μm membrane filter (Millipore Japan, Tokyo, Japan) and sterilized, and organic acid concentrations were measured using a high-performance liquid chromatography (HPLC) system (432 Conductivity Detector; Waters Co., Milford, MA, USA). Meanwhile, the pH of each sample was measured using an IQ 150 pH/Thermometer (IQ Scientific Instruments, Inc., Carlsbad, CA, USA).

### Statistical analyses

Data are expressed as the means ± standard deviations (SD) of normally distributed data, and as the medians (interquartile range) of data with skewed distributions. The Mann-Whitney *U* test was used for data analysis. Detection rates were analyzed using the Fisher exact probability test. False discovery rates (FDR; *q* value) for multiple comparisons of bacterial counts and of organic acid concentrations were calculated using the Benjamini and Hochberg method. Multivariable linear regression analysis was performed to investigate the association between bacterial counts/organic acid concentrations and independent variables, including ischemic stroke, age, and risk factors that differed significantly between patients and controls. Variables were checked for collinearity using the variance inflation factor. All the variance inflation factors were <2, suggesting that multicollinearity was not a problem in this study. Pearson’s correlation coefficients were calculated to detect associations between fecal bacterial counts/organic acid concentrations and bioclinical variables (serum biomarkers, body mass index, age, and NIHSS). Statistical analyses were performed using JMP 12.0.1 software (SAS Inc., Cary, NC, USA).

## Results

### Demographic profiles of study participants

The rates of hypertension (*p* = 0.03) and T2D (*p* < 0.001), as well as the levels of HbA1c (*p* = 0.008), LDL cholesterol (*p* = 0.003), and inflammatory markers such as hsCRP (*p* = 0.01) and IL-6 (*p* < 0.001), were significantly higher in patients with stroke than in the control subjects (Table 1).

**Table 1:** Characteristics of the study participants.

	Stroke patients n = 41	Controls n = 40	*p*-value
Male sex	31 [76]	24 [60]	0.13
Age, years	65.4 ± 14.1	67.4 ± 8.9	0.77
BMI (kg/m^2^)	23.5 (21.5–26.0)	23.4 (21.6–26.0)	0.58
Current smoker	13 [32]	6 [15]	0.08
Hypertension	23 [56]	13 [33]	0.03
Type 2 diabetes	21 [50]	6 [15]	<0.001
Dyslipidemia	22 [54]	18 [45]	0.44
Previous ischemic stroke	2 [5]	0 [0]	0.16
Previous CAD	2 [5]	0 [0]	0.16
Medications			
Aspirin	2 [5]	4 [10]	0.38
Statin	7 [17]	10 [25]	0.38
ARB	8 [20]	7 [15]	0.82
PPI	3 [7]	6 [15]	0.27
HbA1c (%)	6.4 (5.6–7.9)	5.7 (5.4–6.2)	0.008
HDL cholesterol (mg/dl)	52.1 ± 12.4	54.5 ± 13.6	0.42
LDL cholesterol (mg/dl)	133.2 ± 39.4	109.7 ± 25.8	0.003
Triglycerides (mg/dl)	127.0 (85.0–199.0)	133.0 (85.5–192.8)	0.79
WBC (/μl)	7107 ± 2449	5945 ± 1511	0.02
hsCRP (mg/dl)	0.11 (0.04–0.25)	0.06 (0.01–0.09)	0.01
IL-6 (pg/ml)	2.5 (1.7–3.4)	1.6 (1.3–2.5)	<0.001
TNF-α (pg/ml)	1.0 (0.9–1.5)	1.1 (0.8–1.4)	0.54
LBP (μg/ml)	9.0 (6.7–11.9)	8.2 (6.7–10.4)	0.27

ARB, angiotensin-receptor blockers; BMI, body mass index; CAD, coronary artery disease; HbA1c, glycated hemoglobin A1c; HDL, high-density lipoprotein; hsCRP, high sensitivity C-reactive protein; IL, interleukin; LBP, lipopolysaccharide-binding protein; LDL, low-density lipoprotein; PPI, proton pump inhibitor; TNF, tumor necrosis factor; WBC, white blood cell count. Continuous variables are presented as means ± standard deviations or as medians (interquartile range). Categorical variables are presented as absolute numbers [%].

### Composition of fecal bacteria

Total fecal bacterial counts were similar between patients (10.5 ± 0.4 log_10_ cells/g feces) and controls (10.5 ± 0.5 log_10_ cells/g feces), and were not statistically significantly different between groups (*p* = 0.85). Moreover, while there were lower fecal numbers of the *C*. *coccoides* group and the *L*. *sakei* subgroup, and higher numbers of the *Atopobium* cluster and *Enterococcus* among stroke patients than in the control subjects, these differences did not remain statistically significant after FDR adjustment (Table 2, S1A and S1B Fig). Notably, however, the bacterial counts of the *L*. *ruminis* subgroup were significantly higher among *Lactobacillus* species (*p* = 0.003, *q* = 0.02) in patients with stroke than in the controls (S2 Table, S1C Fig).

**Table 2:** Comparisons of fecal bacterial counts between stroke patients and control subjects.

	Bacterial counts (log_10_ cells/g)				Detection rate (%)^a^		
	Stroke patients^b^	Controls^b^	*p*-value^c^	*q*^d^	Stroke patients	Controls	*p-*value^e^
**Obligate anaerobes**							
*C*. *coccoides* group	9.7 ± 0.6	10.0 ± 0.6	0.03	0.13	100	100	1.00
*C*. *leptum* subgroup	9.6 ± 0.6	9.6 ± 0.8	0.81	0.89	100	100	1.00
*B*. *fragilis* group	8.8 ± 0.8	9.2 ± 0.8	0.05	0.13	100	100	1.00
*Bifidobacterium*	9.1 ± 1.3	9.3 ± 0.9	0.93	0.84	100	100	1.00
*Atopobium* cluster	9.5 ± 0.6	9.2 ± 0.5	0.01	0.13	100	95	0.24
*Prevotella*	7.3 ± 1.6	7.1 ± 1.6	0.63	0.84	71	68	0.81
*C*. *difficile*	4.8 ± 0.4	5.3 ± 1.2	1.00	0.84	5	5	1.00
*C*. *perfringens*	9.7 ± 0.6	9.7 ± 0.6	0.97	0.89	56	53	0.82
**Facultative anaerobes**							
*Lactobacillus*	7.4 ± 1.6	7.0 ± 1.4	0.18	0.36			1.00
*Enterobacteriaceae*	7.1 ± 1.0	7.2 ± 1.2	0.71	0.84	100	90	0.05
*Enterococcus*	6.8 ± 1.3	6.1 ± 1.4	0.03	0.13	90	93	1.00
*Streptococcus*	9.3 ± 0.8	9.0 ± 1.0	0.23	0.36	100	98	0.49
*Staphylococcus*	4.7 ± 1.0	4.3 ± 0.7	0.08	0.13	93	90	0.71
**Aerobes**							
*Pseudomonas*	4.6 ± 1.4	4.7 ± 0.7	0.91	0.89	27	33	0.63

^a^Detection rate represents the percentage of fecal samples that contained specific bacterial groups/genera/species above the detection threshold.

^b^Means and standard deviations are indicated.

^c^Statistical differences were examined using the Mann-Whitney *U* test.

^d^*q* values were calculated using the Benjamini and Hochberg method.

^e^Statistical differences were analyzed using Fisher’s exact test.

Because the frequencies of hypertension and T2D differed significantly between the patients with stroke and the control subjects, multivariable linear regression analysis was subsequently performed to screen for associations between bacterial counts and independent variables such as ischemic stroke, hypertension, T2D, and age. Notably, ischemic stroke was associated with increased bacterial counts of the *Atopobium* cluster (*β* = 0.307, *p* = 0.01) and *L*. *ruminis* (*β* = 0.325, *p* = 0.04), and decreased counts of the *L*. *sakei* subgroup (*β* = -0.279, *p* = 0.04), independent of age, hypertension, and T2D. Meanwhile, T2D was associated with decreased counts of *C*. *coccoides* (*β* = -0.298, *p* = 0.01) (Table 3). No associations were detected for the other bacterial species/groups.

**Table 3:** Multivariable linear regression analysis to identify contributors to bacterial counts.

	Bacterial count			
	*C*. *coccoides*	*Atopobium* cluster	*L*. *ruminis* subgroup	*L*. *sakei* subgroup
	β (*p*-value)	β (*p*-value)	β (*p*-value)	β (*p*-value)
Age	-0.270 (0.01)	-0.251 (0.03)	0.099 (0.48)	0.064 (0.62)
Ischemic stroke	-0.132 (0.27)	0.307 (0.01)	0.325 (0.04)	-0.279 (0.04)
Hypertension	-0.072 (0.51)	-0.112 (0.33)	0.200 (0.167)	0.008 (0.95)
Type 2 diabetes	-0.298 (0.01)	-0.118 (0.33)	0.130 (0.40)	0.029 (0.84)

β indicates the standardized regression coefficient.

### Correlations between fecal bacterial counts and metabolic marker levels

We next screened for correlations between bacterial counts or organic acid concentrations and bioclinical markers. To avoid possible drug interactions, patients (n = 27) and controls (n = 36) not taking hypoglycemic drugs were used to investigate correlations with HbA1c level. This analysis included seven patients with stroke and T2D, as their T2D diagnosis was obtained following admission; these individuals were not treated with hypoglycemic drugs. Stroke patients (n = 34) and controls (n = 30) not taking lipid-lowering agents were used to investigate correlations with the levels of TG, LDL cholesterol, and HDL cholesterol. Several microbes correlated with bioclinical markers (Table 4). Among microbes for which bacterial counts were significantly associated with ischemic stroke, the *L*. *ruminis* subgroup count positively correlated with IL-6 levels. Meanwhile, bacterial counts of the *C*. *coccoides* group, which were significantly associated with T2D, were negatively correlated with the levels of HbA1c, LDL cholesterol, and inflammatory markers such as hsCRP and IL-6. No associations were observed between bacterial counts and NIHSS.

**Table 4:** Significant correlations between bacterial counts and bioclinical markers, as represented by Pearson’s correlation coefficient *r* values.

	TG	HbA1c	LDL-C	HDL-C	hsCRP	IL-6	TNF-α	WBC	BMI	Age
**Obligate anaerobes**										
*C*. *coccoides* group		-0.300*	-0.285*		-0.247*	-0.301**				
*Bacteroides fragilis* group						-0.230*				
*Atopobium* cluster										-0.246*
*Prevotella*	-0.287*		-0.376*							
**Facultative anaerobes**										
*L*. *gasseri* subgroup					-0.235*					
*L*. *fermentum*						0.332*				
*L*. *plantarum* subgroup			0.347*							
*L*. *reuteri* subgroup				-0.287*					0.265*	
*L*. *ruminis* subgroup						0.327*				
*Enterobacteriaceae*			-0.270*				0.225*			
*Enterococcus*						0.242*				
*Streptococcus*				-0.307*						
*Staphylococcus*		0.382**								
**Aerobes**										
*Pseudomonas*								0.576**	0.445*	

BMI, body mass index; HbA1c, glycated hemoglobin A1c; HDL, high-density lipoprotein; hsCRP, high sensitivity C-reactive protein; IL, interleukin; LDL, low-density lipoprotein; TG, triglycerides; TNF, tumor necrosis factor; WBC, white blood cells;

*p < 0.05,

**p < 0.01.

### Detection rate of gut bacterial rRNA in the blood of stroke patients and control subjects

Because gut bacteria have been detected in the blood of patients with T2D [14], we screened for the presence of bacteria in the blood of our subjects. The minimum detectable number for all target bacteria by qRT-PCR was one bacterial cell per 1 ml of blood [25]. Gut bacteria were detected in three (7.5%) controls and in two (4.9%) stroke patients, with no significant difference in the prevalence (*p* = 0.62). rRNA of the *C*. *coccoides* group, *Clostridium leptum* subgroup, *Atopobium* cluster, *Prevotella* spp., *L*. *sakei* subgroup, *Enterobacteriaceae*, *Staphylococcus* spp., and *Pseudomonas* spp. was detected in the blood of the controls, whereas only that of *Streptococcus* spp. was detected in the blood of stroke patients. There were no differences in bioclinical variables such as T2D prevalence and inflammatory marker levels between the bacteremic and non-bacteremic subjects (S3 Table).

### Correlation between fecal organic acid concentrations and metabolic and inflammatory marker levels

The total organic acid concentration was significantly lower (*p* = 0.02) in patients with stroke (87.6 ± 30.6 μmol/g feces) than in the control subjects (102.5 ± 26.3 μmol/g feces). Among the organic acids tested, the concentrations of acetic acid were significantly lower (*p* = 0.003, *q* = 0.02), while the concentrations of valeric acid were significantly higher (*p* = 0.003, *q* = 0.02) in patients with stroke compared to the controls. The detection rates of isovaleric and valeric acids were also significantly higher in patients with stroke (*p* = 0.0003 and *p* = 0.03, respectively) than in the controls (Table 5, S2 Fig). In contrast, there was no difference (*p* = 0.15) in pH among the stroke patients (6.78 ± 0.53) and controls (6.62 ± 0.63).

**Table 5:** Comparison of the concentrations of fecal organic acids between patients with stroke and control subjects.

	Concentration (μmol/g)				Detection rate (%)		
	Stroke patients^b^	Controls^b^	*p-*value^c^	*q*^d^	Stroke patients	Controls	*p-*value^e^
Acetic acid	54.2 ± 20.3	66.5 ± 17.8	0.003	0.02	100	100	1.00
Propionic acid	17.2 ± 6.6	19.3 ± 7.2	0.28	0.28	100	100	1.00
Butyric acid	10.1 ± 6.1	9.9 ± 7.5	0.60	0.93	100	100	1.00
Isovaleric acid	2.6 ± 1.5	1.9 ± 1.6	0.02	0.19	95	63	0.0003
Valeric acid	2.0 ± 1.1	1.3 ± 0.8	0.003	0.02	83	60	0.03
Succinic acid	0.8 ± 1.2	3.5 ± 10.2	0.40	0.24	85	78	0.40
Formic acid	1.2 ± 1.6	0.9 ± 1.0	0.07	0.51	95	90	0.43
Lactic acid	1.0 ± 0.7	5.3 ± 11.9	0.79	0.51	12	23	0.25

^a^Detection rate represents the percentage of fecal samples that contained specific bacterial groups/genera/species above the detection threshold.

^b^Means and standard deviations are indicated.

^c^Statistical differences were examined using the Mann-Whitney *U* test.

^d^*q* values were calculated using the Benjamini and Hochberg method.

^e^Statistical differences were analyzed using Fisher’s exact test.

**Fig 1:**
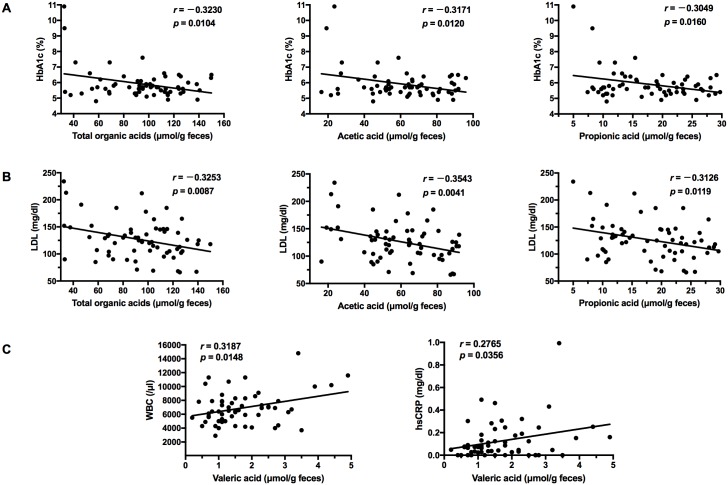
Scatter diagrams of organic acid concentrations and metabolic/inflammatory markers. Correlations between organic acid concentrations and (A) glycated hemoglobin A1c (HbA1c) and (B) low-density lipoprotein (LDL), and (C) between white blood cell counts (WBC) and high sensitivity C-reactive protein (hsCRP) levels were analyzed using Pearson’s correlation.

Notably, multivariable linear regression analysis demonstrated that the acetic acid (*β* = -0.262, *p* = 0.04) and valeric acid (*β* = 0.340, *p* = 0.01) concentrations were independently associated with ischemic stroke (Table 6). However, no associations were detected for the other organic acids. Meanwhile, changes in total organic acids, acetic acid, and propionic acid concentration negatively correlated with HbA1c and LDL cholesterol levels (Fig 1A and 1B). In contrast, changes in the concentration of valeric acid were positively correlated with WBC counts and hsCRP levels (Fig 1C). No associations were observed between the concentrations of other organic acids and bioclinical markers.

**Table 6:** Multivariable linear regression analysis to identify contributors to organic acid concentrations.

	Organic acid concentration			
	Total organic acid	Acetic acid	Butyric acid	Valeric acid
	β (*p*-value)	β (*p*-value)	β (*p*-value)	β (*p*-value)
Age	0.070 (0.53)	0.033 (0.76)	0.164 (0.16)	-0.284 (0.03)
Ischemic stroke	-0.237 (0.06)	-0.262 (0.04)	-0.017 (0.89)	0.340 (0.01)
Hypertension	0.050 (0.67)	0.013 (0.91)	-0.027 (0.82)	0.051 (0.70)
Type 2 diabetes	-0.064 (0.60)	-0.128 (0.30)	0.124 (0.33)	-0.035 (0.80)

β indicates the standardized regression coefficient.

## Discussion

The results of the present study demonstrate that ischemic stroke was associated with certain changes in fecal bacterial counts and with the concentrations of certain organic acids, even after adjustment for stroke risk factors such as hypertension, T2D, and age. Furthermore, these alterations were associated with changes in the levels of inflammatory markers, as well as changes in glucose and lipid profiles.

*Firmicutes* and *Bacteroidetes* constitute the predominant phyla of the human gut, and the proportions of these organisms have a significant relevance to host metabolism [10, 11, 13]. Metagenomic sequencing studies have revealed the diversity of human gut microbiota and the abundance of particular groups/species, particularly that of the predominant organisms. However, the gut microbiota is highly complex and diverse on a more refined level. Notably, the rRNA-targeted qRT-PCR method can be used to quantify the abundance of targeted bacterial populations, including subdominant groups/species, with high resolution. Moreover, this approach is more sensitive, rapid, and accurate than other methods [23, 24]. Indeed, in the present study, qRT-PCR using group-, genus-, or species-specific primers was used to quantify the abundance of target bacterial species with high sensitivity, which could potentially enable a more accurate understanding of the relationship between a particular bacterium and the host. The six predominant obligate anaerobic bacterial groups analyzed in the present study were previously quantified in four healthy Japanese subjects by YIF-SCAN, whereas total intestinal bacterial counts were enumerated by fluorescence *in situ* hybridization (FISH) analysis [24]. We recently reported that the counts of these six predominant bacterial groups/genera obtained via YIF-SCAN analysis corresponded to 71.3 ± 9.4% (mean and SD) of the total intestinal bacterial count, which correlated well with the counts obtained by FISH [27]. We also reported that the five potential gut pathogens are approximately 10,000-times less prevalent than the six predominant anaerobic groups [24]. Thus, these pathogens are present at subdominant levels and might therefore not be quantified efficiently by routine DNA-based PCR or next generation sequencing methods, owing to the lower sensitivity of these approaches. In this context, we therefore employed YIF-SCAN analysis, a highly sensitive approach (capable of detecting approximately 10^2–3^ cells/g feces) that enables the acquisition of highly precise quantitative data regarding subdominant gut members.

Although only the bacterial counts of *L*. *ruminis* were significantly higher in stroke patients compared to the controls, ischemic stroke was identified as the main contributor to counts of some other bacterial groups in multivariable regression analysis. Increased numbers of the *L*. *ruminis* subgroup were associated with ischemic stroke and were positively correlated with IL-6 levels. *L*. *ruminis* subgroup members have been reported to produce flagellin proteins and induce the production of the proinflammatory cytokine IL-8 in cultured human intestinal epithelial cells [28]. Thus, increased *L*. *ruminis* subgroup counts might contribute to inflammation in stroke patients. Conversely, ischemic stroke was also associated with decreased counts of other *Lactobacillus* species such as the *L*. *sakei* subgroup. It was previously reported that *L*. *sakei* is associated with higher BMI in healthy adults and the elderly [29]. Notably, a significant depletion of *L*. *sakei* was reported in the sinus mucosa of patients with chronic rhinosinusitis. These organisms were found to provide a protective effect against sinus mucosa infection through the competitive inhibition of pathogenic bacteria [30]. Although we did not find any significant associations between the *L*. *sakei* subgroup and the bioclinical markers examined, depletion of these bacteria might be deleterious to intestinal mucosal defense in patients with stroke. We also found a significant association between ischemic stroke and increased numbers of the *Atopobium* cluster, although these bacteria were not associated with bioclinical markers. In comparison, *Collinsella* spp., the predominant member of *Atopobium* cluster, is correlated with lipid metabolism in healthy subjects [31].

In our study, decreased counts of the *C*. *coccoides* group were associated with T2D, which is consistent with the findings of a previous study [14]. In addition, these counts were negatively correlated with the levels of HbA1c, LDL cholesterol, and the inflammatory markers hsCRP and IL-6. The *C*. *coccoides* group belongs to the *Firmicutes* phylum, and represents a major component of the human gut microbiota [32]. Notably, *Clostridium* species, particularly clusters IV and XIVa (also known as the *C*. *leptum* subgroup and *C*. *coccoides* group, respectively) were recently demonstrated to induce colonic regulatory T cells, which exert their immunosuppressive activity through IL-10 production [33]. Moreover, the prevalence of this group is also decreased in patients with inflammatory bowel disease [34]. Collectively, these observations suggest that the *C*. *coccoides* group plays an important role in maintaining host metabolism and controlling inflammation.

The gut microbiota might also be linked to host metabolism and inflammation via the production of organic acids. In this study, reduced fecal acetic acid concentrations were significantly associated with ischemic stroke. Furthermore, concentrations of this acid were negatively correlated with HbA1c and LDL cholesterol levels. Meanwhile, although propionic acid concentrations were not statistically associated with ischemic stroke, a similar negative correlation was observed. Supplementation with acetic acid has been shown to reduce cholesterol levels in obese and overweight individuals [35]. Both acetic and propionic acids trigger incretin hormone glucagon-like peptide-1 (GLP-1) secretion by activating G protein-coupled free fatty acid receptor 2 (GPR43) in intestinal L cells, with a subsequent increase in glucose tolerance [36]. In addition, acetic acid-mediated GPR43 activation in adipocytes inhibits fat accumulation in adipose tissue and stimulates the metabolism of unincorporated lipids and glucose in other tissues [37]. A metagenome-wide association study to identify T2D-associated markers reported that patients with T2D were characterized by a decreased abundance of butyric acid-producing bacteria, including the *Clostridium* cluster XIVa [13]. Although the present study also showed a significant association between reduced *C*. *coccoides* group counts and T2D, there was no association between fecal butyric acid concentrations and this disease. In this regard, butyric acid production decreases in the distal part of the large intestine [38], potentially impacting fecal butyric acid concentrations.

Furthermore, we found that valeric acid concentrations and the detection rates of isovaleric and valeric acid were significantly higher in patients with stroke. This increased concentration of valeric acid was associated with ischemic stroke and was positively correlated with inflammatory marker levels such as WBC counts and hsCRP levels. Although gut microbes primarily produce short chain fatty acids (acetic acid, propionic acid, and butyric acid) via carbohydrate fermentation, these organisms also produce branched-chain fatty acids (isobutyric acid and isovaleric acid), accompanied by toxic compounds including ammonia during protein fermentation [39]. In experimental models, dietary supplementation with prebiotics has been shown to promote the growth of presumably beneficial bacterial species and to increase short chain fatty acid concentrations while simultaneously reducing the prevalence of potentially harmful bacteria and the production of protein-derived catabolites, such as isobutyric acid, isovaleric acid, and ammonia, in association with modification of gut permeability and inflammation [40]. In addition, probiotic administration reduces isovaleric and valeric acid fecal concentrations in patients with atherosclerosis [41]. *Oscillibacter* produces valeric acid as the major end-product of glucose metabolism [42]. Notably, patients suffering stroke and transient ischemic attack exhibited higher proportions of *Oscillibacter* than control subjects [18]. In addition, *Oscillibacter* is positively correlated with gut permeability, which can cause host inflammation [43]. Thus, *Oscillibacter* might be harmful to stroke patients; however, these bacteria were not analyzed in our study. The main producer of valeric acid is currently unknown, and it remains unclear whether *Oscillibacter* is the cause of the increased valeric acid concentrations observed in stroke patients in this study.

Elevated blood levels of lipopolysaccharide (LPS), a major component of the outer membrane of gram-negative bacteria, are associated with systemic inflammation and cardiovascular disease events [44]. In a diabetic, high-fat diet mouse model, changes in the composition of the gut microbiota, in the context of increased intestinal permeability, caused subsequent LPS translocation from the gut into the blood, inducing systemic inflammation [45]. While significant increases in the LBP associated with inflammatory marker levels, as well as higher rates of live gut bacteria in the blood, have been reported in patients with T2D [14], we observed no differences in LBP concentrations and bacteremia rates between the patients with stroke and the control subjects.

There are several limitations to the present study. First, causal relationships cannot be inferred from our data. Although we detected an association between the composition of the gut microbiota and risk factors for stroke, information regarding the pre-stroke gut microbial composition of these patients was unavailable. It is therefore possible that the stress associated with stroke itself impacts the gut microbiota [46]. Experimental studies have reported that acute brain ischemia with a large infarct volume can cause gut dysbiosis via stress-induced intestinal dysfunction in mice [47, 48]. In particular, such changes in the gut microbiota involved a significant decrease in the levels of *Prevotellaceae* and an increase in the levels of *Peptococcaceae* in one study [47], whereas overgrowth with a preferential expansion of the *Bacteroidetes* phylum was observed in another [48]. Here, the patient cohort consisted of individuals having experienced relatively less severe stroke (mean NIHSS on admission, 3.2 ± 2.8). Although the microbial changes reported in mice were not observed in our patients, we cannot exclude the possibility that brain injury can have substantial effects on gut microbiota composition. No associations between bacterial counts or organic acid concentrations and NIHSS scores were identified in the present study. Therefore, changes in these factors do not appear to be merely consequential to stroke severity. Although we do not know the origin of these changes, earlier studies demonstrated a link between diet and gut microbial composition [10] and organic acid concentrations [20]. In this regard, dietary changes after admission might also affect gut microbiota in patients with stroke. In addition, the prevalence of T2D was significantly higher in patients with stroke than in control subjects, and a reduction in the prevalence of the *C*. *coccoides* group was associated with T2D in our study. Thus, dysbiosis in patients with stroke appears, at least in part, to be associated with T2D. However, changes in the numbers of the *Atopobium* cluster, *L*. *ruminis*, and *L*. *sakei*, and in acetic and valeric acid concentrations were independently associated with ischemic stroke after multivariable regression analysis. Therefore, gut dysbiosis might be directly or indirectly (by cardiovascular risk factors; e.g., T2D) linked to ischemic stroke. Another limitation of our study consisted of a lack of data regarding the prevalence of metabolic syndrome, which has been associated with gut dysbiosis [49]. Finally, we did not examine the influence of fasting conditions on serum parameters.

In conclusion, we detected a significant association between ischemic stroke and both fecal bacterial counts and organic acid concentrations; these changes were in turn associated with the levels of metabolic and inflammatory biomarkers. The current findings suggest that gut dysbiosis in patients with ischemic stroke is associated with host metabolism and inflammation. Further studies are needed, however, to determine whether these alterations might serve as a predictive indicator of ischemic stroke.

## Supporting information

S1 Fig Scatter plots of fecal bacterial counts in ischemic stroke patients and control subjects. (A) Obligate anaerobes; (B) facultative anaerobes and aerobes; (C) *Lactobacillus*. Means and standard deviations are indicated.
(TIFF)

S2 Fig Scatter plots of concentrations of fecal organic acids in ischemic stroke patients and control subjects. Means and standard deviations are indicated.
(TIFF)

S1 Table 16S and 23S rRNA gene-targeted primers used in this study. (DOCX)

S2 Table Comparisons of fecal *Lactobacillus* counts between ischemic stroke patients and control subjects. (DOCX)

S3 Table Demographic profiles of subjects with and without bacteremia. (DOCX)
