# Correction: Gut dysbiosis is associated with metabolism and systemic inflammation in patients with ischemic stroke

**DOI:** 10.1371/journal.pone.0176062

**Published:** 2017-04-13

**Authors:** Kazuo Yamashiro, Ryota Tanaka, Takao Urabe, Yuji Ueno, Yuichiro Yamashiro, Koji Nomoto, Takuya Takahashi, Hirokazu Tsuji, Takashi Asahara, Nobutaka Hattori

There are values missing from the detection rate column in Table 2. Please see the corrected Table 2 here.

**Table 2:** Comparisons of fecal bacterial counts between stroke patients and control subjects.

	Bacterial counts (log_10_ cells/g)				Detection rate (%)^a^		
	Stroke patients^b^	Controls^b^	*p*-value^c^	*q*^d^	Stroke patients	Controls	*p-*value^e^
**Obligate anaerobes**							
*C*. *coccoides* group	9.7 ± 0.6	10.0 ± 0.6	0.03	0.13	100	100	1.00
*C*. *leptum* subgroup	9.6 ± 0.6	9.6 ± 0.8	0.81	0.89	100	100	1.00
*B*. *fragilis* group	8.8 ± 0.8	9.2 ± 0.8	0.05	0.13	100	100	1.00
*Bifidobacterium*	9.1 ± 1.3	9.3 ± 0.9	0.93	0.84	100	100	1.00
*Atopobium* cluster	9.5 ± 0.6	9.2 ± 0.5	0.01	0.13	100	95	0.24
*Prevotella*	7.3 ± 1.6	7.1 ± 1.6	0.63	0.84	71	68	0.81
*C*. *difficile*	4.8 ± 0.4	5.3 ± 1.2	1.00	0.84	5	5	1.00
*C*. *perfringens*	9.7 ± 0.6	9.7 ± 0.6	0.97	0.89	56	53	0.82
**Facultative anaerobes**							
*Lactobacillus*	7.4 ± 1.6	7.0 ± 1.4	0.18	0.36	100	100	1.00
*Enterobacteriaceae*	7.1 ± 1.0	7.2 ± 1.2	0.71	0.84	100	90	0.05
*Enterococcus*	6.8 ± 1.3	6.1 ± 1.4	0.03	0.13	90	93	1.00
*Streptococcus*	9.3 ± 0.8	9.0 ± 1.0	0.23	0.36	100	98	0.49
*Staphylococcus*	4.7 ± 1.0	4.3 ± 0.7	0.08	0.13	93	90	0.71
**Aerobes**							
*Pseudomonas*	4.6 ± 1.4	4.7 ± 0.7	0.91	0.89	27	33	0.63

^a^Detection rate represents the percentage of fecal samples that contained specific bacterial groups/genera/species above the detection threshold.

^b^Means and standard deviations are indicated.

^c^Statistical differences were examined using the Mann-Whitney *U* test.

^d^*q* values were calculated using the Benjamini and Hochberg method.

^e^Statistical differences were analyzed using Fisher’s exact test.
