# Supplementary figures and images for: Gut dysbiosis is associated with metabolism and systemic inflammation in patients with ischemic stroke
Source: PLoS One. 2017 Feb 6;12(2):e0171521. doi: 10.1371/journal.pone.0171521 (PMC5293236; doi:10.1371/journal.pone.0171521)

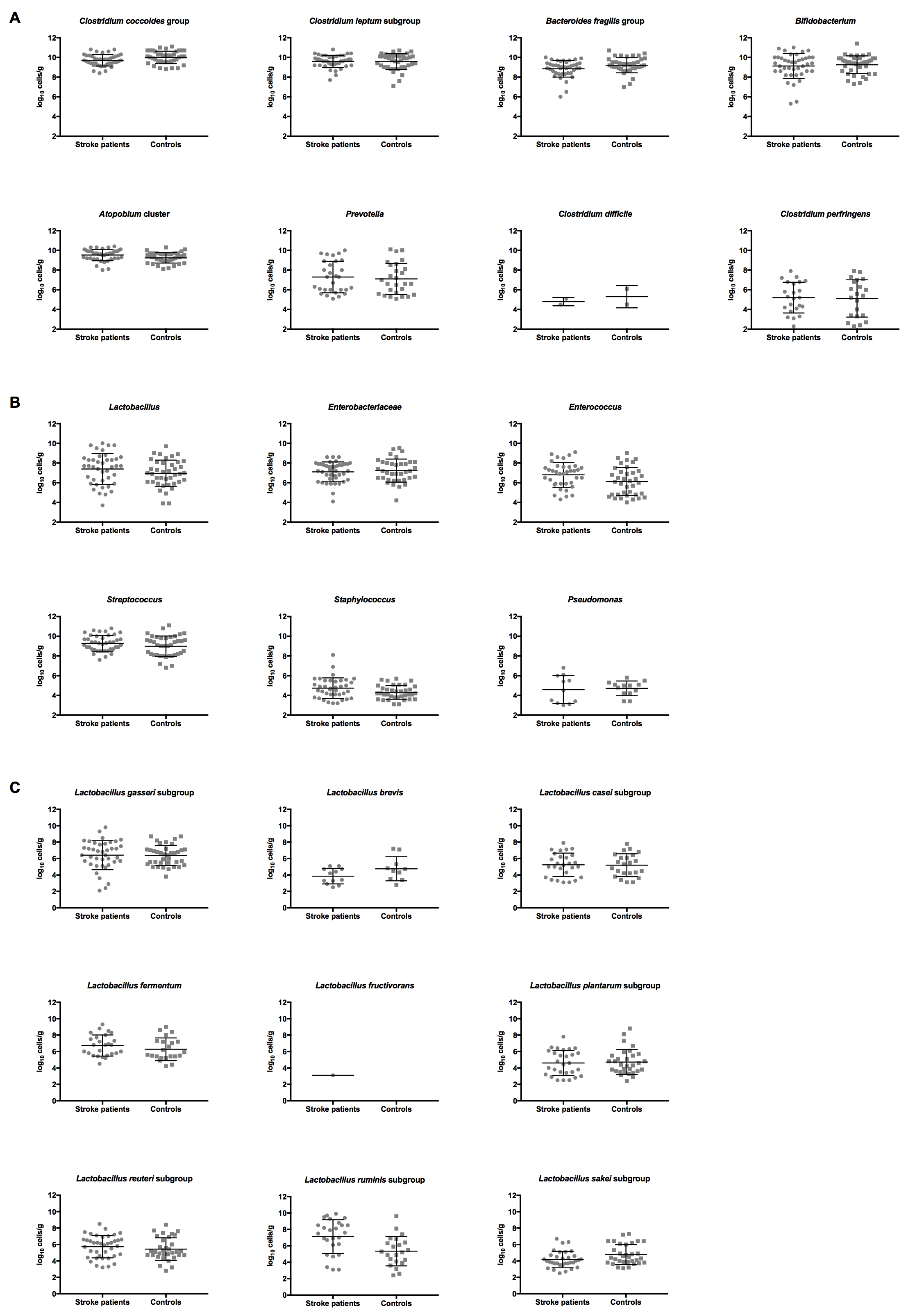

Supplement: S1 Fig — (A) Obligate anaerobes; (B) facultative anaerobes and aerobes; (C) Lactobacillus. Means and standard deviations are indicated. (TIFF) [file pone.0171521.s001.tiff]

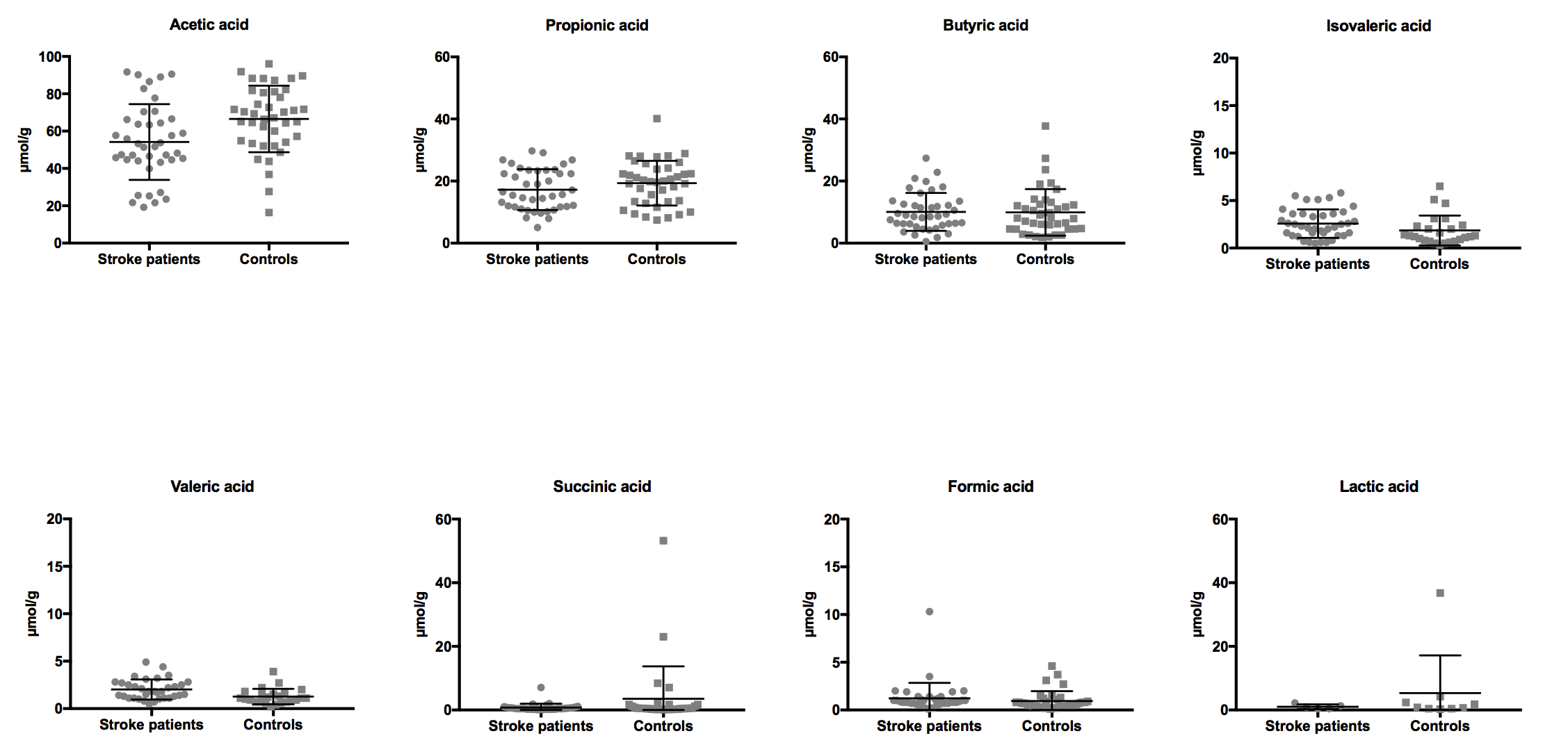

Supplement: S2 Fig — Means and standard deviations are indicated. (TIFF) [file pone.0171521.s002.tiff]
