# Supplementary material for: Gut dysbiosis is associated with metabolism and systemic inflammation in patients with ischemic stroke
Source: PLoS One. 2017 Feb 6;12(2):e0171521. doi: 10.1371/journal.pone.0171521 (PMC5293236; doi:10.1371/journal.pone.0171521)
Supplement: S1 Table — (DOCX) [file pone.0171521.s003.docx]

**S1 Table. 16S and 23S rRNA gene-targeted primers used in this study**

| Target bacteria* | Primer | Sequence (5’ - 3’) | Ref |
| --- | --- | --- | --- |
| *Clostridium coccoides* group | g-Ccoc-F | AAATGACGGTACCTGACTAA | 6 |
|  | g-Ccoc-R | CTTTGAGTTTCATTCTTGCGAA |  |
| *Clostridium leptum* subgroup | sg-Clept-F | GCACAAGCAGTGGAGT | 7 |
|  | sg-Clept-R3 | CTTCCTCCGTTTTGTCAA |  |
| *Bacteroides fragilis* group | g-Bfra-F2 | AYAGCCTTTCGAAAGRAAGAT | 8 |
|  | g-Bfra-R | CCAGTATCAACTGCAATTTTA | 6 |
| *Bifidobacterium* | g-Bifid-F | CTCCTGGAAACGGGTGG | 6 |
|  | g-Bifid-R | GGTGTTCTTCCCGATATCTACA |  |
| *Atopobium* cluster | g-Atopo-F | GGGTTGAGAGACCGACC | 7 |
|  | g-Atopo-R | CGGRGCTTCTTCTGCAGG |  |
| *Prevotella* | g-Prevo-F | CACRGTAAACGATGGATGCC | 6 |
|  | g-Prevo-R | GGTCGGGTTGCAGACC |  |
| *Clostridium difficile* | Cd-lsu-F | GGGAGCTTCCCATACGGGTTG | 4 |
|  | Cd-lsu-R | TTGACTGCCTCAATGCTTGGGC |  |
| *Clostridium perfringens* | s-Clper-F | GGGGGTTTCAACACCTCC | 2 |
|  | ClPER-R | GCAAGGGATGTCAAGTGT | 5 |
| *Lactobacillus casei* subgroup | sg-Lcas-F | ACCGCATGGTTCTTGGC | 2 |
|  | sg-Lcas-R | CCGACAACAGTTACTCTGCC |  |
| *Lactobacillus gasseri* subgroup | sg-Lgas-F | GATGCATAGCCGAGTTGAGAGACTGAT | 2 |
|  | sg-Lgas-R | TAAAGGCCAGTTACTACCTCTATCC |  |
| *Lactobacillus plantarum* subgroup | sg-Lpla-F | CTCTGGTATTGATTGGTGCTTGCAT | 2 |
|  | sg-Lpla-R | GTTCGCCACTCACTCAAATGTAAA |  |
| *Lactobacillus reuteri* subgroup | sg-Lreu-F | GAACGCAYTGGCCCAA | 2 |
|  | sg-Lreu-R | TCCATTGTGGCCGATCAGT |  |
| *Lactobacillus ruminis* subgroup | sg-Lrum-F | CACCGAATGCTTGCAYTCACC | 2 |
|  | sg-Lrum-R | GCCGCGGGTCCATCCAAAA |  |
| *Lactobacillus sakei* subgroup | sg-Lsak-F | CATAAAACCTAMCACCGCATGG | 2 |
|  | sg-Lsak-R | TCAGTTACTATCAGATACRTTCTTCTC |  |
| *Lactobacillus brevis* | s-Lbre-F | ATTTTGTTTGAAAGGTGGCTTCGG | 2 |
|  | s-Lbre-R | ACCCTTGAACAGTTACTCTCAAAGG |  |
| *Lactobacillus fermentum* | LFer-1 | CCTGATTGATTTTGGTCGCCAAC | 2 |
|  | LFer-2 | ACGTATGAACAGTTACTCTCATACGT |  |
| *Lactobacillus fructiborans* | s-Lfru-F | TGCGCCTAATGATAGTTGA | 2 |
|  | s-Lfru-R | GATACCGTCGCGACGTGAG |  |
| *Enterobacteriaceae* | En-lsu-3F | TGCCGTAACTTCGGGAGAAGGCA | 1 |
|  | En-lsu-3'R | TCAAGGACCAGTGTTCAGTGTC |  |
| *Enterococcus* | g-Encoc-F | ATCAGAGGGGGATAACACTT | 2 |
|  | g-Encoc-R | ACTCTCATCCTTGTTCTTCTC |  |
| *Staphylococcus* | g-Staph-F | TTTGGGCTACACACGTGCTACAATGGACAA | 2 |
|  | g-Staph-R | AACAACTTTATGGGATTTGCWTGA |  |
| *Streptococcus* | g-Str-F | AGCTTAGAAGCAGCTATTCATTC | 3 |
|  | g-Str-R | GGATACACCTTTCGGTCTCTC |  |
| *Pseudomonas* | PSD7F | CAAAACTACTGAGCTAGAGTACG | 1 |
|  | PSD7R | TAAGATCTCAAGGATCCCAACGGCT |  |

*Specific primer sets were developed by using 16S rDNA sequences, except for Cd-lsu-F/R, En-lsu-3F/3’R, and g-Str-F/R, which targeted 23S rDNA.

**References**

1. Matsuda K, Tsuji H, Asahara T, Kado Y, Nomoto K. Sensitive quantitative detection of commensal bacteria by rRNA-targeted reverse transcription-PCR. Appl Environ Microbiol 2007;73: 32-39.
2. Matsuda K, Tsuji H, Asahara T, Matsumoto K, Takada T, Nomoto K. Establishment of an analytical system for the human fecal microbiota, based on reverse transcription-quantitative PCR targeting of multicopy rRNA molecules. Appl Environ Microbiol 2009;75: 1961-1969.
3. Sakaguchi S, Saito M, Tsuji H, Asahara T, Takata O, Fujimura J, et al. Bacterial rRNA-targeted reverse transcription-PCR used to identify pathogens responsible for fever with neutropenia. J Clin Microbiol 2010;48: 1624-1628.
4. Matsuda K, Tsuji H, Asahara T, Takahashi T, Kubota H, Nagata S, et al. Sensitive quantification of *Clostridium difficile* cells by reverse transcription-quantitative PCR targeting rRNA molecules. Appl Environ Microbiol 2012;78: 5111-5118.
5. Kikuchi E, Miyamoto Y, Narushima S, Itoh K. Design of species specific primers to identify 13 species of *Clostridium* harbored in human intestinal tracts. Microbiol Immunol 2002;46: 353–358.
6. Matsuki T, Watanabe K, Fujimoto J, Miyamoto Y, Takada T, Matsumoto K, et al. Development of 16S rRNA-gene-targeted group-specific primers for the detection and identification of predominant bacteria in human feces. Appl Environ Microbiol 2002;68: 5445-5451.
7. Matsuki T, Watanabe K, Fujimoto J, Takeda T, Tanaka R. Use of 16S rRNA gene-targeted group-specific primers for real-time PCR analysis of predominant bacteria in human feces. Appl Environ Microbiol 2004;70: 7220-7228.
8. Matsuki T. Development of quantitative PCR detection method with 16S rRNA gene-targeted genus- and species-specific primers for the analysis of human intestinal microflora and its application. Nihon Saikingaku Zasshi 2007;62: 255-261. [Article in Japanese]
