# Supplementary material for: Gut dysbiosis is associated with metabolism and systemic inflammation in patients with ischemic stroke
Source: PLoS One. 2017 Feb 6;12(2):e0171521. doi: 10.1371/journal.pone.0171521 (PMC5293236; doi:10.1371/journal.pone.0171521)
Supplement: S2 Table — (DOCX) [file pone.0171521.s004.docx]

**S2 Table. Comparisons of fecal *Lactobacillus* counts between stroke patients and control subjects.**

|  | Bacterial counts (log_10_ cells/g) | | | | Detection rate (%)^a^ | | |
| --- | --- | --- | --- | --- | --- | --- | --- |
|  | Stroke patients^b^ | Controls^b^ | *p*-value^c^ | *q*^d^ | Stroke patients | Controls | *p*^e^ |
| *L. gasseri* subgroup | 6.4 ± 1.8 | 6.4 ± 1.2 | 0.90 | 0.90 | 98 | 93 | 0.34 |
| *L. brevis* | 3.9 ± 1.0 | 4.8 ± 1.5 | 0.13 | 0.26 | 29 | 25 | 0.80 |
| *L. casei* subgroup | 5.2 ± 1.4 | 5.2 ± 1.4 | 0.90 | 0.90 | 66 | 58 | 0.50 |
| *L. fermentum* | 6.7 ± 1.3 | 6.3 ± 1.4 | 0.24 | 0.49 | 63 | 55 | 0.50 |
| *L. fructivorans* | 3.1 | <2.3 | — | — | 2 | 0 | 1.00 |
| *L. plantarum* subgroup | 4.6 ± 1.5 | 4.7 ± 1.5 | 0.78 | 0.90 | 68 | 78 | 0.46 |
| *L. reuteri* subgroup | 5.7 ± 1.4 | 5.4 ± 1.4 | 0.37 | 0.59 | 95 | 83 | 0.09 |
| *L. ruminis* subgroup | 7.1 ± 2.1 | 5.4 ± 1.8 | 0.003 | 0.02 | 63 | 55 | 0.50 |
| *L. sakei* subgroup | 4.2 ± 1.0 | 4.8 ± 1.2 | 0.04 | 0.12 | 80 | 80 | 1.00 |

^a^Detection rate represents the percentage of fecal samples that contained specific bacterial groups/genera/species above the detection threshold.

^b^Mean and standard deviations are indicated

^c^Statistical differences were examined using the Mann-Whitney *U* test.

^d^*q* values were calculated using the Benjamini and Hochberg method.

^e^Statistical differences were analyzed using Fisher’s exact test.
