# Supplementary material for: Gut dysbiosis is associated with metabolism and systemic inflammation in patients with ischemic stroke
Source: PLoS One. 2017 Feb 6;12(2):e0171521. doi: 10.1371/journal.pone.0171521 (PMC5293236; doi:10.1371/journal.pone.0171521)
Supplement: S3 Table — (DOCX) [file pone.0171521.s005.docx]

**S3 Table. Demographic profiles of subjects with and without bacteremia.**

|  | With bacteremia | Without bacteremia | *p*-value |
| --- | --- | --- | --- |
|  | n = 5 | n = 76 |  |
| Male sex | 4 [80] | 51 [67] | 0.55 |
| Age, years | 64.6 ± 5.4 | 66.5 ± 12.1 | 0.32 |
| BMI (kg/m^2^) | 20.6 (16.8‐23.3) | 23.5 (21.6‐26.1) | 0.08 |
| Current smoker | 2 [40] | 17 [22] | 0.37 |
| Hypertension | 4 [80] | 41 [54] | 0.26 |
| Type 2 diabetes | 4 [80] | 50 [66] | 0.51 |
| Dyslipidemia | 4 [80] | 52 [68] | 0.59 |
| Previous ischemic stroke | 0 [0] | 2 [3] | 0.71 |
| Previous CAD | 0 [0] | 2 [3] | 0.71 |
| Medications |  |  |  |
| Aspirin | 1 [20] | 5 [7] | 0.27 |
| Statin | 0 [0] | 17 [22] | 0.23 |
| ARB | 0 [0] | 15 [20] | 0.27 |
| PPI | 0 [0] | 9 [12] | 0.41 |
| HbA1c (%) | 5.6 (5.0‐6.3) | 5.9 (5.5‐6.6) | 0.25 |
| HDL cholesterol (mg/dl) | 53.0 ± 12.1 | 53.3 ± 13.1 | 0.94 |
| LDL cholesterol (mg/dl) | 132.8 ± 54.3 | 120.9 ± 34.0 | 0.88 |
| Triglycerides (mg/dl) | 196.0 (100.0‐303.5) | 127.0 (84.3‐184.5) | 0.29 |
| WBC (/µl) | 6200 ± 954 | 6555 ± 2166 | 0.94 |
| hsCRP (mg/dl) | 0.20 (0‐0.28) | 0.07 (0.03‐0.17) | 0.67 |
| IL-6 (pg/ml) | 1.9 (1.2‐6.9) | 2.0 (1.4‐2.8) | 0.67 |
| TNF-α (pg/ml) | 1.1 (0.9‐2.5) | 1.0 (0.8‐1.4) | 0.52 |
| LBP (µg/ml) | 9.5 (5.9‐15.4) | 8.6 (6.7‐11.2) | 0.84 |

ARB, angiotensin-receptor blockers; BMI, body mass index; CAD, coronary artery disease; HbA1c, glycated hemoglobin A1c; HDL, high-density lipoprotein; hsCRP, high sensitivity C-reactive protein; IL, interleukin; LBP, lipopolysaccharide-binding protein; LDL, low-density lipoprotein; PPI, proton pump inhibitor; TNF, tumor necrosis factor; WBC, white blood cell count. Continuous variables are presented as means ± standard deviations or as medians (interquartile range). Categorical variables are presented as absolute numbers [%].
